# Immunotherapeutic Potential of Eugenol Emulsion in Experimental Visceral Leishmaniasis

**DOI:** 10.1371/journal.pntd.0005011

**Published:** 2016-10-24

**Authors:** Mohammad Islamuddin, Garima Chouhan, Muzamil Yaqub Want, Hani A. Ozbak, Hassan A. Hemeg, Farhat Afrin

**Affiliations:** 1 Parasite Immunology Laboratory, Department of Biotechnology, Jamia Hamdard (Hamdard University), New Delhi, India; 2 Department of Medical Laboratories Technology, Faculty of Applied Medical Sciences, Taibah University, Medina, Kingdom of Saudi Arabia; Yale School of Public Health, UNITED STATES

## Abstract

**Background:**

The therapy of visceral leishmaniasis (VL) is limited by resistance, toxicity and decreased bioavailability of the existing drugs coupled with dramatic increase in HIV-co-infection, non-availability of vaccines and down regulation of cell-mediated immunity (CMI). Thus, we envisaged combating the problem with plant-derived antileishmanial drug that could concomitantly mitigate the immune suppression of the infected hosts. Several plant-derived compounds have been found to exert leishmanicidal activity via immunomodulation. In this direction, we investigated the antileishmanial activity of eugenol emulsion (EE), complemented with its immunomodulatory and therapeutic efficacy in murine model of VL.

**Methodology/Principal Findings:**

Oil-in-water emulsion of eugenol (EE) was prepared and size measured by dynamic light scattering (DLS). EE exhibited significant leishmanicidal activity with 50% inhibitory concentration of 8.43±0.96 μg ml^-1^ and 5.05±1.72 μg ml^─1^, respectively against the promastigotes and intracellular amastigotes of *Leishmania donovani*. For *in vivo* effectiveness, EE was administered intraperitoneally (25, 50 and 75 mg/kg b.w./day for 10 days) to 8 week-infected BALB/c mice. The cytotoxicity of EE was assessed in RAW 264.7 macrophages as well as in naive mice. EE induced a significant drop in hepatic and splenic parasite burdens as well as diminution in spleen and liver weights 10 days post-treatment, with augmentation of 24h-delayed type hypersensitivity (DTH) response and high IgG2a:IgG1, mirroring induction of CMI. Enhanced IFN-γ and IL-2 levels, with fall in disease-associated Th2 cytokines (IL-4 and IL-10) detected by flow cytometric bead-based array, substantiated the Th1 immune signature. Lymphoproliferation and nitric oxide release were significantly elevated upon antigen revoke *in vitro*. The immune-stimulatory activity of EE was further corroborated by expansion of IFN-γ producing CD4^+^ and CD8^+^ splenic T lymphocytes and up-regulation of CD80 and CD86 on peritoneal macrophages. EE treated groups exhibited induction of CD8^+^ central memory T cells as evidenced from CD62L and CD44 expression. No biochemical alterations in hepatic and renal enzymes were observed.

**Conclusions:**

Our results demonstrate antileishmanial activity of EE, potentiated by Th1 immunostimulation without adverse side effects. The Th1 immune polarizing effect may help to alleviate the depressed CMI and hence complement the leishmanicidal activity.

## Author Summary

In the absence of vaccines and escalating resistance to the available chemotherapeutic drugs, alternate therapeutic options are urgently needed for visceral leishmaniasis (VL) or kala-azar, a systemic and potentially fatal, vector-borne neglected tropical disease, caused by *Leishmania donovani*. In the present study, we explored the two-prong effect of eugenol emulsion (EE) in eliminating the parasites with synergistic boosting of the dampened immune system, characteristic of active disease. Infected BALB/c mice upon intraperitoneal administration of EE, exhibited a significant decline in liver and spleen parasite load with concomitant drop in splenomegaly. Protection in treated mice coincided with Th1 immunopotentiation as was evident from substantial DTH and lymphoproliferative responses, elevated levels of IgG2a over IgG1 isotypes, significant enhancement in IFN-γ producing CD4^+^ and CD8^+^ T cells, waning levels of IL-4 and IL-10, augmented nitrite levels, induction of immunological memory and stimulation of antigen presenting capacity of macrophages, compared to infected mice. The dual antileishmanial and immunostimulatory potential of EE with no adverse toxic effects validates it as an adjunct to chemotherapy that may aid in leishmanicidal activity via ameliorating the depressed cellular immunity.

## Introduction

Leishmaniasis, a complex vector-borne parasitic syndrome, is caused by obligate intra-macrophagic protozoan parasites of the genus *Leishmania*, a member of the order Kinetoplastida. The array of manifestations varies from a self-limiting cutaneous form to a potentially lethal visceralizing infestation of the liver, spleen and bone marrow [1]. Resolution of disease correlates with *Leishmania-*specific CD4^+^-type 1 T helper (Th1) and CD8^+^ T lymphocyte responses with production of interferon-γ (IFN-γ), macrophage nitric oxide (NO) and reactive oxygen species (ROS) generation [2–3]. *Leishmania* species exploit discrete mechanisms to elude the cellular immune defenses, such as inhibition of phagolysosomal fusion, and reactive nitrogen species (RNS)- and ROS-mediated macrophage microbicidal effects, dampening of cell-mediated immune response via blockade of antigenic peptide display to T cells, impaired secretion of Th1 cytokines, and infiltration of IL-10 producing T regulatory cells [4–6]. The treatment of visceral leishmaniasis (VL) is complicated because of intra-macrophagic refuge of the amastigotes, rendering the patient immunodeficient and unable to eliminate the parasites through the natural defense mechanisms [7]. The quandary of VL has been compounded due to concomitant infection in acquired immunodeficiency syndrome (AIDS) patients [8].

There is no vaccine available against VL though several are in phase III clinical trials [9]. The existing anti-leishmanial therapy suffers from grave impediments such as drug resistance, compromising efficacy, toxicity, prolonged courses and parenteral routes of administration [10–12]. Hence new drugs for the treatment of VL are imperative. In an ongoing quest for safe and cheap antileishmanial agents, plant-based secondary metabolites are gaining ground [13–15]. The use of plant products as immune-stimulants has a traditional history. Treatment of leishmaniasis with natural or synthetic molecules appears to be dependent upon the development of an effective immune response that activates macrophages and lymphocytes to release their effector molecules [16–17].

A plethora of studies have reported immunomodulation with plant secondary metabolites such aslicarin A, [18], niranthrin [19], alkaloid skimmianine [20], quassin [21], tannins and structurally related compounds [22], N-Palmitoyl-S-(2,3-bis(palmitoyloxy)-(2RS)-propyl)-Cys-Ser-Lys4 hydrochloride (Pam3Cys) [23] and linalool component of essential oil [24]. Synergistic antileishmanial and immunopotentiating effects of plant fractions or compounds have also been documented [25]. This may result in enhanced clearance of the parasites coupled with boosting of the depressed immunity associated with active VL.

**Fig 1:**
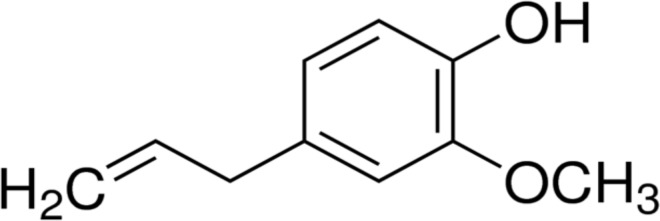
Structure of Eugenol (4-Allyl-2-methoxyphenol)

Eugenol (Fig 1) is the major constituent of *Syzygium aromaticum*. *S aromaticum* or common clove, is indigenous to tropical America and Australia [26] and is endowed with antibacterial [27] and anti-trypanosomal activities [28]. The extracts from flower buds of *S*. *aromaticum* has been reported to display antimalarial efficacy [29]. The immunomodulatory effect of *S*. *aromaticum* essential oil has been attributed in augmentation of humoral and cell mediated immune responses [30]. We have previously evaluated the leishmanicidal effect of eugenol-rich essential oil of *S*. *aromaticum* against promastigotes and intramacrophagic-amastigotes of *L*. *donovani* [31]. However, the poor solubility and high volatility limits its stability resulting in paradigm shift from therapeutic use of most oils to biocompatible emulsifiers. The antibacterial and antifungal activities of eugenol emulsions have been explored [32–33]. Encouraged by the above studies, we evaluated the antileishmanial and immunomodulatory potential of eugenol emulsion (EE) against experimental VL in BALB/c mice.

## Materials and Methods

Eugenol, RPMI 1640 medium, M-199 medium, penicillin G sodium salt, streptomycin sulphate, 3-{4,5-dimethylthiazol-2-yl}-2,5-diphenyltetrazolium bromide (MTT), carboxyfluoresceinsuccinimidyl ester (CFSE), AmB, anti-mouse IgG and isotype antibodies, o-phenylenediaminedihydrochloride (OPD) were procured from Sigma-Aldrich (St Louis, MO, USA). Fetal bovine serum (FBS) was obtained from Gibco-BRL, DMSO from SRL, methanol from Merck, limulus amebocyte lysate (LAL) kit from Pierce, Thermo Scientific. Fluorochrome conjugated anti-mouse antibodies such as CD4-phycoerythin (PE), CD8-fluorescein isothiocyanate (FITC), CD80-allophycocyanin (APC), CD86-phycoerythin cyanine dye 7, APC-CD4 and PE-CD8, FITC-IFN-γ, CD8-APC, CD62L-PE and CD44-FITC, isotype controls and Brefeldin A and cytokine bead array kit (CBA) were procured from BD Pharmingen, USA. Aspartate aminotransaminase (AST), alanine transaminase (ALT), alkaline phosphatase (ALP), creatinine and urea kits were purchased from Span Diagnostics Ltd (Surat, Gujarat, India). Besides these, the analytical grade reagents were used.

### Ethics Statement

The experiments were performed on female BALB/c mice, aged about 6–8 weeks (20–25 g) while the *L*. *donovani* parasites were maintained in Syrian golden hamsters (4–6 weeks old), after prior assessment and approval of the study protocol (Ethics Clearance number 459) by the Jamia Hamdard Animal Ethics Committee (JHAEC). JHAEC is registered under the Committee for the purpose of control and supervision of experiments on animals (CPCSEA) that is registered under Animal Welfare Division of the Central Government of India. The animals were kept in the Central Animal House facility of Hamdard University as per the CPCSEA guidelines. The animals were housed in standard size polycarbonate cages in groups of at least 5 mice (or 3 hamsters) per cage, with temperature maintained at 23 ± 1°C, relative humidity (55 ± 10%), 12:12 h light: dark cycle with *ad libitum* access to a standard pellet diet (Ashirwad Industries, Chandigarh, India) and drinking water. For *ex vivo* experiments, RAW 264.7 cells were procured from National Centre for Cell Science, Pune, India. Undesirable side effects of EE for painful abdominal distention and the resulting distress have been avoided by daily monitoring. We have used 0.15 ml for the intraperitoneal injection of EE in mice, that is, below the recommended volume (0.2 ml) for intraperitoneal administration of emulsion in mice.

### *Leishmania* culture and maintenance

*Leishmania donovani* (MHOM/IN/AG/83) promastigotes were cultured in M199 medium, supplemented with 10% heat-inactivated FBS, 2 mM glutamine, 100 units/ml penicillin G sodium, and 100 μg/ml streptomycin sulphate at 22°C. The parasites were maintained in culture for 4–5 days (initial inocula being 1X10^6^ parasites/ml) and the late log or stationary phase promastigotes were harvested after the second or third passage.

### Cell culture

Murine macrophage-like cells, RAW 264.7 were maintained at 37°C in 10% FBS-supplemented RPMI-1640 medium (pH 7.4) for 48–72 h in a humidified atmosphere with 5% CO_2_. The sub-confluent cultures (70–80%) were split in fresh medium split at 2 x 10^5^ cells ml^─1^.

### Formulation and characterization of Eugenol emulsion

Eugenol was procured from Sigma-Aldrich and oil-in-water emulsion (EE) was prepared using non-ionic surfactant, Tween 80 (T-80) and water to achieve a final concentration of oil mixture in the emulsion as 200 mg ml^─1^. Emulsion was formulated by mixing Eugenol and surfactant, 4% micellar solution was prepared with T-80 and deionized water. Eugenol was poured into the micellar solution through calibrated micropipette under continuous stirring. The formulated emulsion was analyzed for particle size by dynamic light scattering (DLS) Nano-S90 (Nanoseries, Malvern Instruments, UK). The samples were measured [34] at 25°C with a fixed angle of 90°. The dosing emulsion was carefully stored in aluminium foil covered-glass vials at 4° C prior to biological assays.

### Dose-dependent anti-promastigote activity for determination of IC_50_

Promastigotes (2 x 10^6^ cells ml^─1^) were incubated for 72 h at 22°C without or with serial three-fold dilutions of EE, starting from 100 μg ml^─1^ (100, 33.33, 11.11, 3.70, 1.23 and 0.41 μg ml^─1^). The mean percent (%) viability was calculated by MTT assay [35]. The inhibitory concentration responsible for 50% reduction in promastigote growth (IC_50_), was graphically extrapolated by plotting percent (%) viability versus drug concentration [31]. Surfactant, T-80 (0.00175%, present in 100 μg ml^─1^ EE) was used as negative control.

### *Ex vivo* anti-amastigote activity and NO release from macrophages

To assess the effects of EE on intracellular *L*. *donovani* amastigotes, RAW 264.7 macrophages (1X10^6^ cells ml^─1^) were infected with parasites in late log or stationary phase (cell/promastigote ratio, 1/10) at 37°C for 24 h. Thereafter, the non-ingested parasites were gently aspirated, and the infested macrophages were further incubated with EE (0–100 μg ml^─1^) and with negative control (surfactant) for 48 h. The cells were then fixed, Giemsa-stained and microscopically evaluated for percent amastigote infectivity. At least 200 macrophages were enumerated per coverslip, and the concentration of EE that reduced amastigote infectivity by 50% (IC_50_) was calculated.

In parallel, the nitrite concentration in the cell culture supernatants was analysed using Griess reaction. Briefly, to the harvested supernatant, an equal volume of Griess reagent (0.1% N-{1-naphthyl} ethylenediaminedihydrochloride and 1% sulphanilamide in 5% phosphoric acid) was added. After incubation for 10 min at room temperature (RT), the absorbance values were read at 550 nm, and nitrite concentration was calculated by performing linear regression of a standard curve of sodium nitrite [36]. EE and all the reagents were analyzed for endotoxin, lipopolysaccharide (LPS) by chromogenic Limulus Amoebocyte Lysate (LAL) kit according to the manufacturer’s instructions (Pierce, Thermo Scientific) and were found to be free of LPS (0.2 ng ml^─1^ endotoxin).

### MTT cytotoxicity assay *ex vivo*

To determine the adverse cytotoxic effects of EE, RAW 264.7 cells in RPMI 1640 medium were incubated for 48 h at 37°C in a humidified 5% CO_2_ incubator with increasing concentrations of EE (0–200 μg ml^─1^). Surfactant was used as negative control. MTT assay was used to evaluate cell viability, expressed as a percentage relative to untreated macrophages as control [35].

### *In vivo* antileishmanial potential of EE in *L*. *donovani* infected BALB/c mice

Six to eight weeks old BALB/c mice were injected with 2×10^7^ stationary phase *L*. *donovani* promastigotes in the lateral tail vein. At eight weeks of infection, after confirming the parasite load in three randomly selected animals; the mice were arbitrarily divided into four groups of 10 animals each (A- D). Group A comprised of untreated infected mice (INF); Group B–Saline administered vehicle control mice (VC, i.p). Groups C, D, E (EE, i.p.), Infected mice that received respectively three doses of EE (25/50/75 mg/kg body weight {b.w.}) each day for 10 days. Group F- treated with Amphotericin B (AMB, 5 mg/kg b.w., i.v., alternately over a ten day period), worked as the positive control. Ten days post-treatment, mice were euthanized by carbon dioxide asphyxiation for enumeration of hepatic and splenic amastigote burden that was expressed in terms of Leishman Donovan Units (LDU). LDU was evaluated per organ from giemsa-stained multiple impressions smears [23] as the number of amastigotes per 500 host cells X organ weight (mg). Percent reduction in amastigote load (% protection) was calculated as the difference between LDU of infected control and treated mice/ LDU of infected control X 100. Protection coincided with a drop in hepato-splenomegaly and parasite clearance with respect to untreated infected controls [37].

### Freeze-thawed (FT) and soluble leishmanial antigen (SLA) preparation

SLA and FT antigens were prepared from promastigotes in the stationary-phase [38–39]. Briefly, promastigotes in third or fourth passage were harvested, washed four times in cold 1X PBS and resuspended at 2x10^8^ cells ml^─1^. The suspension was frozen at ─80°C (30 min) and thawed in a 37°C water bath (15 min) alternately for 6 cycles. For SLA preparation, after ten alternate freeze-thawing cycles, the suspension was centrifuged (5250 x g, 4°C, 10 min) and the supernatant containing leishmanial antigens (SLA) harvested. FT and SLA were stored at ─70°C until use and the protein quantitated [40].

### Induction of delayed-type hypersensitivity (DTH)

DTH response in infected mice subsequent to treatment was evaluated as a hallmark of cellular immunity. Briefly, two days prior to euthanisation, mice were intradermally injected with FT (50 μl: 800 μg ml^─1^) in the right footpad and PBS in the left footpad. After 48 h, the thickness of footpads was recorded using vernier calipers and the results expressed as the difference in swelling of the right compared to the contralateral left hind footpad [39, 41].

### Determination of serum IgG1 and IgG2a isotypes

The *Leishmania-*specific serum IgG subclasses were measured through enzyme-linked immunosorbent assay (ELISA). In brief, FT (0.25 μg/well) was seeded in the wells of ELISA plates (Nunc, Roskilde, Denmark) for 1 h at 37°C. After three washes, blocking was done with 1% BSA for 2 h at RT followed by addition of 1,000-fold diluted mice sera. Post-washing, IgG1 and IgG2a isotype-specific goat anti-mouse secondary antibodies (Sigma Aldrich) were added and the plates incubated at 37°C for 1 h. After washing, incubation with peroxidase-conjugated rabbit anti-goat IgG as the tertiary antibody (Sigma Aldrich) was done at 37°C for 1 h. Post-washing, OPD was added and the absorbance taken on an ELISA plate reader at 490 nm [41].

### Nitrite estimation

Culture supernatants of peritoneal macrophages from normal and infected mice following treatment were analyzed for LPS- and SLA-specific nitrite (NO_2_) levels by the Griess method as described previously [42]. Briefly, to the culture supernatants, equal volume of Griess reagent was added and incubated for 15 min at RT. The optical density (OD) was measured at 550 nm using a microplate reader. Dilute solution of sodium nitrite (NaNO_2_) in culture medium served as a standard. All the reagents and fractions were free of LPS (0.2 ng/ml endotoxin) as confirmed by LAL assay.

### Lymphocyte proliferation

To assess the effect of EE on the proliferation of lymphocytes, single cell suspension from spleens (5 × 10^6^ cells ml^─1^) and lymph nodes (2 × 10^6^ cells ml^─1^) of treated and untreated mice were seeded into 96-well microplates (200 μl/well) and *in vitro* stimulated with SLA (10 μg ml^─1^) at 37°C for 48 h in a humid-saturated atmosphere containing 5% CO_2._ Lymphoproliferation was assessed by enumerating the cells using a hemocytometer [43]. Alternatively, for tracking lymphoproliferation by CFSE dilution, the SLA (10 μg ml^-1^)-stimulated lymphocytes (5 ×10^6^ cells ml^-1^) from infected, treated and naïve mice were labeled with 1 μM CFSE for 48 h. After washing twice with PBS, the cells were resuspended in PBS and acquired in a BD LSR II flow cytometer to assess the population of cells that underwent proliferation. The contour plots were generated after appropriate gating [44].

### Analysis of Th1/Th2 cytokines

Cytokine concentrations (IL-12, IL-4, IL-10 and IFN-γ) in the serum and splenocyte culture supernatants of differently treated mice were estimated by a multiplex bead-based assay as per the instructions of the manufacturer. [45–46]. Briefly, culture supernatants, serum samples and the cytokine standards were added in equal volumes to antibody-coated capture beads prior to incubation with biotinylated detection antibodies (anti-mouse) for 1 h at RT in the dark. The beads were washed with wash buffer (400 x g, 4°C, 5 min) and the supernatant gently aspirated. The beads were washed twice followed by incubation for a period of 1 h at RT in the dark with streptavidin-PE. After performing two additional centrifugation steps as described above, the beads were re-suspended in assay buffer and acquired on a BD LSR II flow cytometer (Becton Dickinson). The data were analysed with BD CBA software based on standard curves generated with recombinant cytokines.

### Lymphocyte phenotyping

CD4^+^ and CD8^+^ T cell phenotyping was performed as previously described [47]. Splenocytes from treated and untreated BALB/c mice were co-stained with anti-CD4 FITC and anti-CD8 PE antibodies for 15 min on ice. The cells were then washed and resuspended in PBS for acquisition on a BD LSR II flow cytometer equipped with DIVA software.

### Detection of intracellular IFN-γ levels

Intracellular detection of IFN-γ- producing CD4^+^ and CD8^+^ T lymphocytes was performed by flow cytometry. Single cell suspensions from spleens of treated and untreated infected mice were *in vitro* stimulated with 10 μg ml^-1^ SLA for 24 h and further incubated with Brefeldin A (10 μg ml^-1^) for 1 h. After washing with FACS buffer, the cells were co-stained with APC and PE conjugated anti-CD4 and anti-CD8 antibodies, respectively. This was followed by washing, fixing and permeabilization with BD Cytofix/Cytoperm. The cells were then stained with FITC-anti-IFN-γor isotype-matched control monoclonal antibodies (mAbs), and acquired and analyzed on a flow cytometer. The CD4^+^ and CD8^+^ T lymphocytes were gated individually and the expression of IFN-γ-producing cells ascertained [41].

### Phenotypic analysis of co-stimulatory molecules (CD80 and CD86)

Peritoneal macrophages (2 × 10^6^ cells ml^─1^) from infected BALB/c mice prior or subsequent to treatment were washed with FACS buffer (1X PBS with 1% FBS). To quantify the expression of co-stimulatory molecules, 2 × 10^6^ macrophages from each sample were stained with APC-labeled anti-CD80 (B7-1) and PE-Cy7 conjugated anti-CD86 (B7-2) mAbs on ice for 15 min. The cells were washed twice with PBS and flow cytometric acquisition was performed on BD LSR II equipped with DIVA software [48].

### Analysis of memory CD8^+^ T cells

Splenocytes from infected BALB/c mice prior or subsequent to treatment were washed with FACS buffer and stained with anti-mouse CD8-APC, CD62L-PE and CD44-FITC (BD Pharmingen) for 30 min at 4°C, and then washed and fixed with 2% paraformaldehyde. Cells were acquired on a BD LSR II flow cytometer [49].

### *In vivo* toxicity assay

Ten days post-treatment, blood was drawn from retro-orbital plexus of naïve, infected and treated BALB/c mice and serum separated. To evaluate the hepatic and renal functions, the serum levels of SGOT, SGPT, ALP, urea and creatinine were measured using commercially available kits (Span Diagnostics Ltd.) [39].

### Statistical analyses

All the *in vitro* experiments were repeated at least twice. The *in vivo* data are from five mice per group. Statistical analysis was performed using Graph Pad Prism 5 software. *P* value was calculated using ANOVA with Tukey’s post-test. We considered *P* values <0.05 to be statistically significant. The graphs represent the mean with standard error bars.

## Results

### Eugenol nanoemulsion (EE)

**Fig 2:**
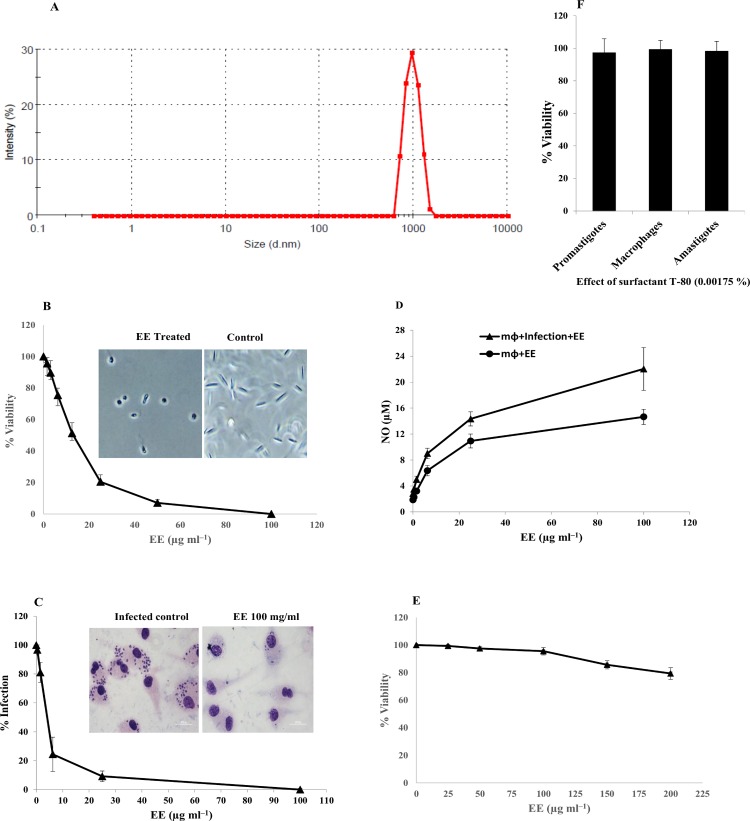
(A) Particle size distribution of eugenol emulsion (EE). Exponential phase *L*. *donovani* promastigotes were incubated at different concentrations of EE (0–100 μg/ml) as described in methods. (B) Peritoneal macrophages after infection, were incubated with increasing concentrations of EE (starting at 100 μg ml^─1^) for 48 h at 37°C: (C) Intracellular amastigotes percentage (%) infectivity. (D & E) NO and superoxide anion generation by uninfected (filled circle) or infected (filled triangle) macrophages post-incubation with EE. Effect of Surfactant (F) on promastigotes, amastigotes and on RAW 264.7 macrophages and adverse effect of EE on RAW 264.7 macrophages ascertained as % viability 48h post-incubation. Each point or bar corresponds to the mean ± SE of triplicate samples and is representative of one of three independent experiments.

The average droplet size and size distribution of eugenol nanoemulsion (EE) was found to be 990.8±2.64 nm and 0.23±0.01, respectively (**Fig 2A**).

### Antileishmanial activity of EE against *L*. *donovani in vitro* and *ex vivo*

#### i) Determination of IC_50_ of EE against promastigotes

The antileishmanial activity of EE was evaluated against *L*. *donovani* promastigotes by MTT reduction assay. EE (0–100 μg ml^-1^) demonstrated dose-dependent killing of the promastigotes and IC_50_ was achieved at 8.43±0.96 μg ml^-1^ (**Fig 2B**). Parasite viability was not affected by the surfactant, T-80 (0.00175%) used as negative control (**Fig 2F**).

#### ii) Antileishmanial effect of EE against intracellular amastigotes

The activity of EE on internalized amastigotes was analyzed microscopically on Giemsa–stained macrophages. Our results depicted a dose-dependent inhibition of EE (0–100μg ml^-1^) on amastigote infectivity with IC_50_ of 5.05±1.72 μg ml^─1^ (**Fig 2C**). At the highest dose of EE (100μg ml^-1^) tested, there was almost complete clearance of the intracellular amastigotes (**Fig 2C**) as revealed from Giemsa-stained micrographs of parasitized macrophages. The amastigote infectivity was not affected by surfactant used as negative control (**Fig 2F**).

### EE induced NO production *ex vivo* from parasitized macrophages

Low basal levels of NO were detected in the cell-free culture supernatants of infected macrophages, correlating with progression of disease. The NO release from infected macrophages upon subsequent incubation with EE (0–100 μgml^-1^) was dose-dependent (**Fig 2D**) and the levels were higher than that produced by normal macrophages. The highest dose of EE (100μg ml^-1^) induced 14.56±1.16 and 20.03±3.28 μM of NO from normal and parasitized macrophages, respectively.

### Cytotoxicity of EE on mammalian macrophages

Exposure of RAW 264.7 to EE (**Fig 2E**) and surfactant **(Fig 2F)** did not compromise the viability and morphology of the murine macrophage cell line.

### Antileishmanial effect of EE *in vivo*

**Fig 3:**
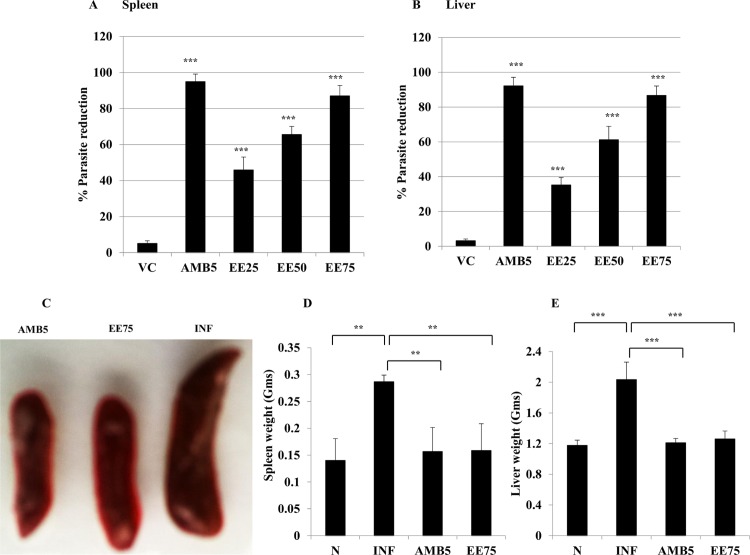
Effect of eugenol emulsion (EE) on established *L*. *donovani* infection. Therapeutic effect of EE (25, 50 and 75mg/kg. bw.) was compared with vehicle control (VC). Percent parasitic reduction in spleen (A) and liver (B) were determined at 10 days post-treatment as defined in Materials and Methods. Size (C) and weight of spleen (D) and liver (E) were also analyzed compared to untreated infected control. Data represent the mean ±SE for five animals per group. Data were tested by ANOVA. Differences between means were assessed for statistical significance by Tukey’s test (**, *P* ≤ 0.01; ***, *P* ≤ 0.001). Results are from one of three representative experiments.

The intra-peritoneal administration of EE (75 mg/kg b.w.) for 10 consecutive days to 8-weeks infected BALB/c mice caused 87.01±5.85% (*P*< 0.001) and 86.68±5.42% (*P*< 0.001) decrease in parasitic load in spleen and liver, respectively (**Fig 3A and 3B**) at 10 days post-treatment. At lower dose of EE (50 mg/kg b.w.), 65.52±4.55% (*P*< 0.001) and 61.19±7.76% (*P*< 0.001) protection were conferred in spleen and liver, respectively. The lowest dose (25 mg/kg b.w.) resulted in more than 45% (*P*< 0.001) fall in hepatic and splenic parasitic burden. AMB (5mg/kg b.w.) induced 92.22±4.96% and 94.88±4.25% elimination of parasites from liver and spleen, respectively. A significant reduction in spleen size (**Fig 3C, inset**) was also observed at 75mg/kg b.w. of EE, compared to the infected control. 50% and 25.68% reduction in spleen and liver weights, respectively was found with EE at this dose (**Fig 3D and 3E**), which was comparable to that obtained with AMB (52.33% and 28.41%).

### EE induced leishmanial antigen-specific DTH

**Fig 4:**
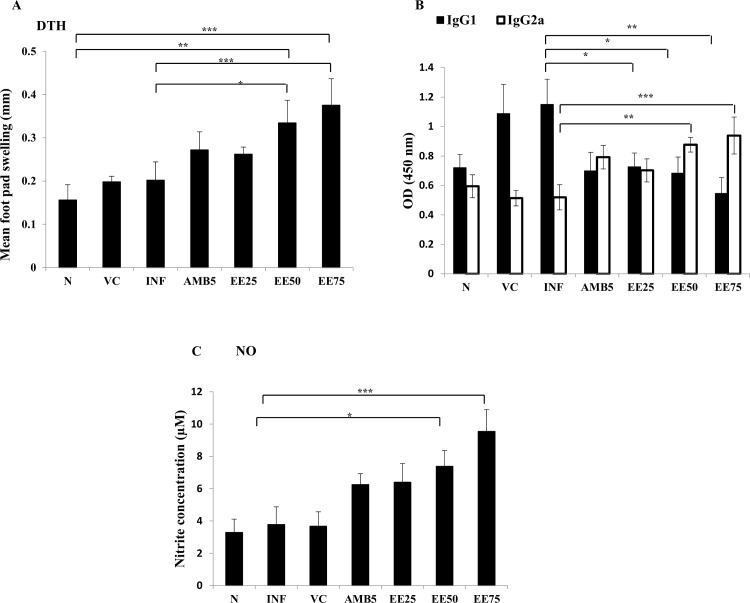
FT-specific DTH and antibody responses and SLA-specific nitrite production in infected mice upon EE treatment compared with the infected control. (A) DTH response was evaluated by measuring the difference in the footpad swelling at 24 h following intradermal inoculation of the test footpad with 50 μl (800 μg/ml) of FT compared with contralateral (PBS-injected) footpad. (B) Sera from treated and control animals were analyzed for FT specific anti-IgG1 and anti-IgG2a levels by ELISA. (C) Macrophages isolated from peritoneal cavity of different groups of mice were stimulated with SLA for 48 h. Nitrite levels in the culture supernatant were determined by the Griess assay. Data represent the mean ±SE for five animals per group. Data were tested by ANOVA. Differences between means were assessed for statistical significance by Tukey’s test (*, *P* ≤ 0.05; **, *P* ≤ 0.01; ***, *P* ≤ 0.001). Results are from one of three representative experiments.

As an *in vivo* correlate of cell-mediated immunity, DTH reaction to FT was measured in infected mice at 10 days post-treatment. EE treatment resulted in a significant enhancement (*P*< 0.001) in footpad thickness at 48 h compared with the control group (0.20±0.02 mm). A dose related increase in DTH reactivity was observed, with maximum swelling at 75 mg/kg b.w. (0.37±0.035 mm) followed by 50 and 25mg/kg b.w. (0.33±0.03 and 0.26±0.01 mm, respectively, **Fig 4A**). Whereas AMB treated mice showed marginal levels (0.27±0.02 mm) of DTH response.

### Humoral immune response in EE-treated mice

At 10 days post-treatment, mice sera were assayed for FT-specific IgG1and IgG2a isotype levels. IgG1 was detected at significantly (*P* ≤ 0.01) higher levels over IgG2a in infected control animals compared with the treated groups; the IgG2a:IgG1 ratio being 0.45 (**Fig 4B**). The highest IgG2a:IgG1ratio (1.72) was observed in mice treated with 75mg/kg b.w. EE (*P*<0.001), followed by 50 mg/kg b.w. EE (1.28), AMB (1.14) and 25 mg/kg b.w. EE (0.97) treatment groups.

### Effect of EE on NO production in protected mice

Host defense against intracellular pathogens including *Leishmania* is primarily mediated by NO and related RNIs [49]. NO production from peritoneal macrophages of EE treated mice was used to evaluate the effectiveness of EE on macrophage microbicidal activity. Nitrite, the stable end-product of NO metabolism was assessed by Griess reagent. Three-fold higher (9.54±0.77 μM) nitrite levels were found in mice treated with higher dose of EE (75mg/kg/b.w., *P*< 0.001) as compared to infected control (3.78±0.63 μM) and the response was dose-dependent (**Fig 4C**). AMB treated mice induced moderate levels of nitrite (6.26± 0.38 μM).

### Lymphoproliferative effect of EE on the splenic and lymph node cells

**Fig 5:**
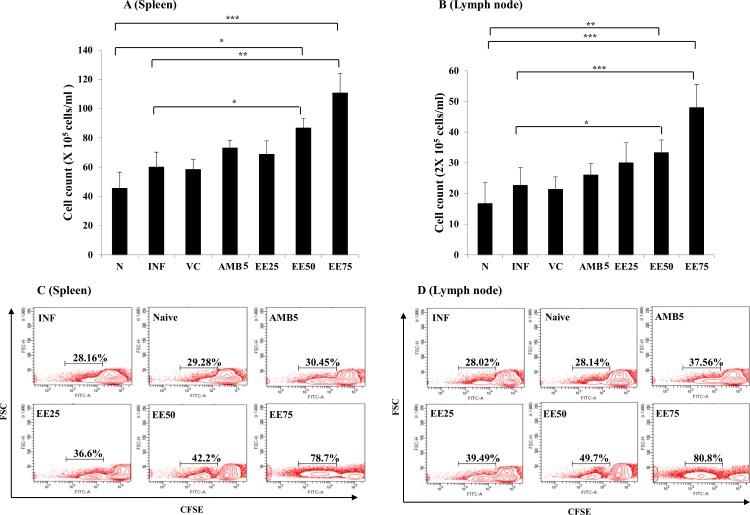
Effect of EE on SLA-specific lymphoproliferation in infected mice following therapy compared with the naïve and infected control. 10 days post-treatment, lymphocytes isolated from (A) spleen (5 × 10^6^ cells ml^─1^) and (B) lymph nodes (2 × 10^6^ cells ml^─1^) were plated aseptically and stimulated with SLA (10μg ml^-1^) for 48 h and enumerated microscopically after trypan blue dye exclusion. In parallel, for lymphoproliferation by CFSE staining, lymphocytes isolated from (C) spleen and (D) lymph nodes were CFSE labeled (in triplicates) and stimulated with SLA for subsequent acquisition and analysis by FACS. Data represent the mean ± SE for five animals per group. Data were tested by ANOVA. Differences between means were assessed for statistical significance by Tukey’s test (*, *P* ≤ 0.05; **, *P* ≤ 0.01; ***, *P* ≤ 0.001). Results are from one of three representative experiments.

The immunomodulatory potential of EE was evaluated through lymphoproliferation. Upon microscopic enumeration, a significant (*P*< 0.001) proliferative effect of SLA-stimulated splenocytes as well as lymphocytes was observed at 10 days post-EE treatment. The lymphoproliferative response was dose-dependent with maximum being elicited at 75mg/kg b.w. that was followed by 50 and 25mg/kg b.w. EE treatment In contrast, AMB did not induce significant SLA-specific lympho-proliferation (**Fig 5A and 5B**).

In parallel, the lymphoproliferative potential of lymphocytes following EE treatment was corroborated by CFSE staining. 28.16% and 28.02% lymphocytes from spleen and lymph nodes, respectively underwent cell division in normal mice. The lymphoproliferative response was the highest in spleen (78.7%) and lymph nodes (80.8%) upon treatment with EE (75 mg/kg. b. w.). In untreated infected control group, we observed 29.28% and 28.14% lymphoproliferation in spleen and lymph nodes, respectively **(Fig 5C and 5D)**.

### Effect of EE on Th1 and Th2 cytokine profile

**Fig 6:**
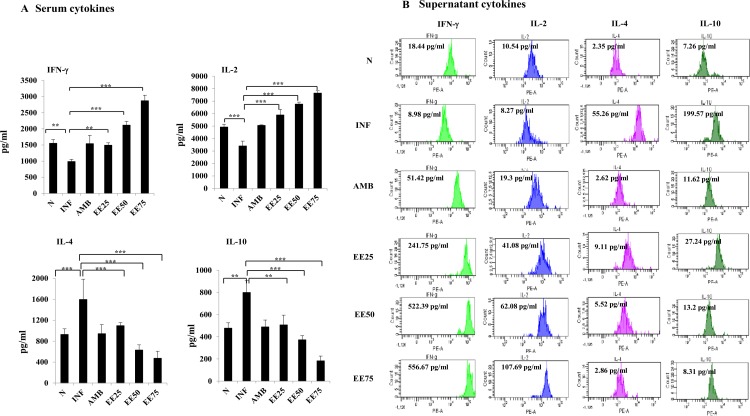
SLA-specific cytokine levels in infected mice upon EE (25, 50 and 75gm/kg. bw.) treatment compared with the untreated infected control. 10 days post-treatment, splenocytes (5 × 10^6^ cells ml^─1^) were plated aseptically and stimulated with SLA (10μg ml^-1^) for 48 h. IFN-γ, IL-2, IL-4 and IL-10 in culture supernatant of *in vitro* restimulated splenocytes (A) and the sera of differently treated and infected mice were measured by bead-based multiplex assay (BD CBA kit) according to the manufacturer’s instructions as elaborated in Methods section. Data are represented as mean ± S.E. of five animals per group. Data were tested by ANOVA. Differences between means were assessed for statistical significance by Tukey’s test (**, *P* ≤ 0.01; ***, *P* ≤ 0.001). Results are from one of three representative experiments.

Changes in the levels of classical Th1 (IFN- γ and IL-2) and Th2 (IL-4 and IL-10) cytokines were assessed on day 10 post-treatment. Compared to untreated infected controls, EE (75mg/kg b.w.) induced enhanced serum levels of IFN-γ and IL-2 (2871±121 and 6943.5±129.77 pgml^─1^, respectively, *P* ≤ 0.001) and significantly lower levels of IL-4 and IL-10 (472.5±96.53 and 182.5±30.76 pgml^─1^, *P* ≤ 0.001) (**Fig 6A**). Whereas, AMB treatment restored the cytokines to normal levels. Similar effect on Th1 and Th2 type cytokines was also observed with the culture supernatants of infected and treated mice (**Fig 6B**).

### EE induced expansion of CD4^+^ and CD8^+^ T cells:

**Fig 7:**
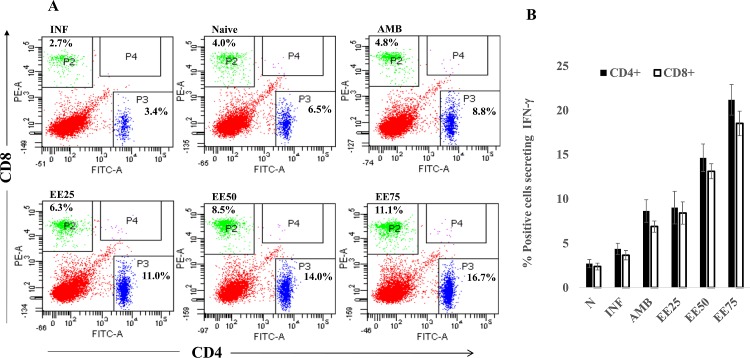
(A) Flow cytometric analysis of CD4^+^ and CD8^+^ T cells of differently treated and untreated infected BALB/c mice. Splenocytes (2 × 10^6^ cells) from 10 days post-treated mice were stained with anti- CD4 FITC and anti- CD8 PE antibodies as described in Methods section. Data are represented as percent CD4^+^ and CD8^+^ T cell populations. (B) Frequency of IFN-γ producing CD4^+^ and CD8^+^ T cells of differently treated and untreated infected BALB/c mice. Splenocytes were stimulated with SLA (10μg ml^-1^). Surface phenotyping and intracellular staining were performed as described in Methods, and the cells were examined by flow cytometry. Mean percentages of CD4^+^ and CD8^+^ T cells producing IFN-γ in each group of untreated and protected BALB/c mice are presented. The significance of differences between the means was determined by Student’s *t* test (***, *P* ≤ 0.001).

Mice with established *L*. *donovani* infection had low numbers of splenic CD4^+^ (7.2%) and CD8^+^ (4.1%) T cells (**Fig 7**) that enhanced to 14.4% and 7.6%, respectively 10 days post-treatment with EE (25 mg/kg b.w.). The T cell counts were increased with 50 mg/kg b.w. EE treatment (17% CD4^+^ and 9.4% CD8^+^ T cells). The CD4^+^ and CD8^+^ T cell population was, however, the highest (18.8% and 10.7%, respectively) at 75 mg/kg b.w. of EE (**Fig 7A**). These data support a bias towards Th1-driven effector functions and the role of CD4^+^ as well as CD8^+^ T cells in protection following EE treatment.

### EE induced high frequency of IFN-γ secreting CD4 and CD8 T cells

CD4^+^ and CD8^+^ T cells are the main cellular sources of IFN-γ. In infected mice, low frequencies of IFN-γ-producing CD4^+^ (4.38±0.35%) and CD8^+^ (3.64±0.30%) T cells were detected which were upregulated by AMB treatment (9.04±0.72% CD4^+^, 6.89±0.36% CD8^+^). The maximum induction of IFN-γ-secreting CD4^+^ and CD8^+^ T cells occurred at 75mg/kg. b.w. of EE (21.18±0.99%, 18.51±0.80%, respectively). While EE at 25mg/kg. b.w. induced 9.02±1.06% CD4^+^ and 8.41±0.72% CD8^+^ T cells secreting IFN-γ that was comparable to that elicited by AMB (**Fig 7B**).

### EE elicited CD80 and CD86 expression on APCs

**Fig 8:**
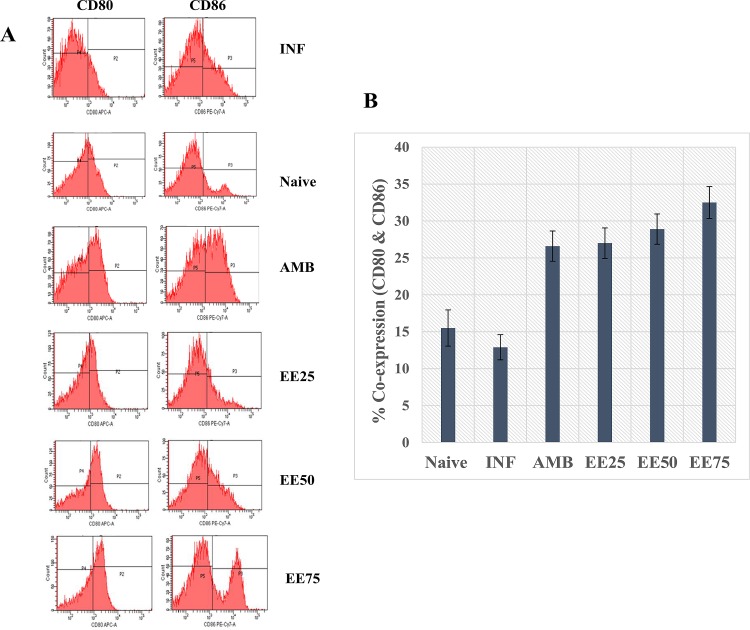
(A) Flow cytometric analysis of the percentage of peritoneal macrophages expressing CD80 and CD86 co-stimulatory signaling molecules in differently treated and untreated infected BALB/c mice. To quantify the expression of co-stimulatory molecules, 2 × 10^6^ macrophages were stained with APC-labeled anti-CD80 (B7-1) and PE-Cy7 labeled anti-CD86 (B7-2) mAbs as described in Methods section. (B) The bar graph represents percent CD80 and CD86 co-expressing cell population. Data represent the mean ± SE for five animals per group. *, *P* ≤ 0.05; **, *P* ≤ 0.01; ***, *P* ≤ 0.001 compared with the control group determined by one way ANOVA. Results are from one of three representative experiments.

The co-stimulatory molecules (CD80 and CD86) on APCs are essential for optimal T-APC talk and lymphocyte activation for secretion of effector cytokines. It was found that EE significantly augmented the expression of both CD80 and CD86 on peritoneal macrophages. EE (25 mg/kg b.w.) optimally boosted the number of double positive cells co-expressing CD80 and CD86 (27%) over the naïve (15.5%) and infected control (12.9%) groups (**Fig 8**). The response was dose-dependent with heightened expression at 50 mg/kg b.w. of EE (28.9%). CD80 and CD86 double positive cells were maximally expressed at 75 mg/kg b.w. EE (32.5%). The response in case of AMB treated group was also enhanced up to a moderate level (**Fig 8**).

### Generation of memory CD8 T (CD44^+^CD62L^+^) lymphocytes

**Fig 9:**
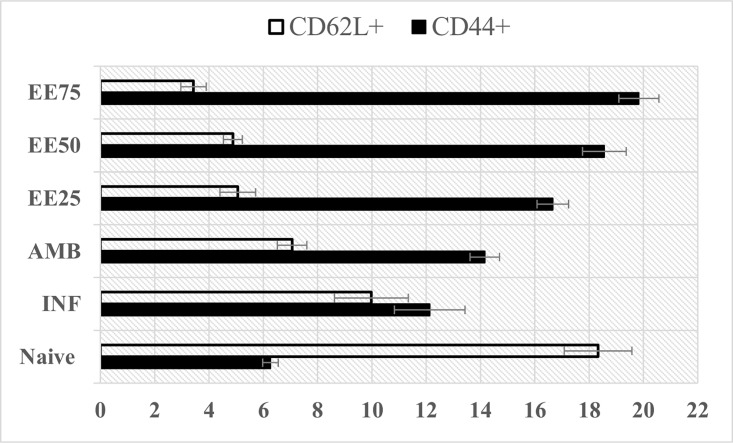
Analysis of CD62L and CD44 expressing memory subsets on splenic lymphocytes isolated from differently treated and infected mice. 2 × 10^6^ lymphocytes were stained with anti-CD8-APC, anti-CD44-FITC and anti-CD62L-PE. Cells were gated on CD8^+^ T lymphocytes. Comparison between the infected vs. EE treated groups is shown. Data represent the mean ± SE for five animals per group. *, *P* ≤ 0.05; **, *P* ≤ 0.01; ***, *P* ≤ 0.001 compared with the control group determined by one-way ANOVA. Results are from one of three representative experiments.

Memory T cell generation in the host is indicative of resistance to *Leishmania* re-infection [50]. The subtle co-expression of CD44 and CD62L (10.7%) in the infected control group, was up-regulated (16.73.6%) upon treatment with EE at 75mg/kg b.w., in concordance with resolution of disease and generation of central memory cells. AMB had insignificant effect (10.93%) on induction of memory T cells **(Fig 9).**

### *In vivo* toxicity of EE by biochemical analysis of serum enzymes:

Liver function (ALP, SGOT and SGPT) and renal function tests (urea and creatinine) were done 10 days post-administration of EE in naive BALB/c mice (**Table 1**) as well as infected mice upon treatment (**Table 2**). EE treated group showed normal values of serum enzymes (up to 75 mg/kg b.w.), indicating no *in vivo* toxicity.

**Table 1:** Effect of EE (higher dose) on hepatic and renal functions of naive BALB/c mice

Group (n = 5)	SGOT (U/L)	SGPT (U/L)	ALP (U/L)	Urea (mg/dL)	Creatinine (mg/dL)
Control	55.40± 5.12	32.09± 10.01	85.06±1.37	16.10± 2.15	0.86± 0.14
EE 75 mg/kg (i.p)	52.9± 3.19	30.98± 7.36	84.16± 6.06	15.49± 3.61	0.85± 0.08

*Mice (n = 5) received* EE *for 10 consecutive days*. *Enzyme estimations (mean± SE) were done using commercial kits*

**Table 2:** Effect of EE on hepatic and renal functions of naïve and *L*. *donovani* infected BALB/c mice upon treatment.

Group (n = 5)	SGOT (U/L)	SGPT (U/L)	ALP (U/L)	Urea (mg/dL)	Creatinine (mg/dL)
Control (Naive)	49.10 ± 6.09	31.16 ± 6.91	86.23 ± 7.33	15.90 ± 4.42	0.98 ± 0.21
EE 75 mg/kg (i.p)	47.09 ± 1.26	30.19 ± 3.19	85.67 ± 4.19	16.66 ± 1.96	1.06 ± 0.07
Infected control	46.29 ± 4.39	30.76 ± 3.11	87.20 ± 2.6	16.40 ± 2.09	1.14 ± 0.39
AMB 5 mg/kg (i.v)	58.63 ± 3.71	37.10 ± 1.14	84.96 ± 3.2	19.16 ± 1.17	0.97 ± 0.13
EE 25 mg/kg (i.p)	44.50 ± 2.30	28.19 ± 4.60	84.06 ± 1.4	16.01 ± 0.89	0.94 ± 0.10
EE 50 mg/kg (i.p)	44.03 ± 3.26	30.21 ± 2.07	85.01 ± 9.1	16.56 ± 1.63	1.05 ± 0.13
EE 75 mg/kg (i.p)	46.69 ± 1.19	31.08 ± 2.16	86.09 ± 3.6	16.99 ± 1.19	1.11 ± 0.10

*Mice (n = 5) received* EE *for 10 consecutive days*. *Enzyme estimations (mean± SE) were done using commercial kit*

## Discussion

The immune system is known to synergistically augment the therapeutic effectualness of anti-parasitic drugs [51]. The administration of immunomodulators in conjunction with conventional chemotherapy to rejuvenate the host immune response has a multitude of benefits and aids in advancing the current therapeutic effectiveness by reducing the dose and duration of treatment and hence the toxicity of the drugs. Thus, antileishmanial drugs that can concomitantly ameliorate the immune suppression of the infected hosts, have a two-prong effect and are sought after [52–56].

Previous report showed that the essential oil of *S*. *aromaticum* (EROSA), mediated programmed cell death against *L*. *donovani* without affecting the host macrophages. Eugenol was the major constituent in EROSA [31]. This is the first report of therapeutic and immunostimulatory potential of emulsion of eugenol (EE) against experimental VL. EE showed profound antileishmanial efficacy, coupled with cell-mediated immunopotentiation without any adverse effect on the host. Furthermore, EE exhibited a concentration-dependent significant antileishmanial activity against *L*. *donovani* promastigotes as well as the intramacrophagic amastigotes. *Ex vivo* studies revealed non-toxicity of EE against murine macrophages even at 100μg ml^─1^. In case of *L*. *amazonensis*, clearance of both axenic and intracellular amastigotes has been attributed to NO [57]. We observed a significant increase in NO (20.03±3.28 μM) post-incubation of amastigote-infested macrophages with EE, harmonizing with the previous reports. The EE-induced NO production from infected macrophages strengthened the role of RNS in amastigote death.

Intra-peritoneal treatment with EE significantly (*P*<0.001) lowered the splenic and hepatic parasite loads in infected mice to levels similar to that achieved with AMB. This was coupled with restoration of liver and spleen weights to normal levels. Our results warrant further studies with lower doses of EE.

Our study corroborates immunomodulation complementing the leishmanicidal effect of EE, apparently through Th1 cytokine-driven and macrophage-mediated mechanisms which are also NO-dependent. Immunostimulatory effects have been reported for several natural products, offering a rational basis for their therapeutic potency. In case of leishmaniasis, an ideal drug is envisaged to have dual effects–to directly and selectively kill the parasites as well as rejuvenate the depressed immunity towards Th1 bias [58, 55].

During the progression of leishmaniasis, cytokine milieu switches from Th1 to Th2 profile [59–60]. An effective therapeutic intervention against *L*. *donovani* entails mounting of a strong Th1 response. To prove that EE has immunomodulatory effect, we analysed the cytokines in serum and culture supernatants of lymphocytes from infected BALB/c mice 10 days post-treatment. Therapy with EE mounted a polarized Th1 response with enhanced IFN-γ and IL-2 secretion, which stimulated microbicidal responses of macrophages leading to NO production that was demonstrated in this study *ex vivo* as well as *in vivo*. Both reactive oxygen and nitrogen species have been implicated to contribute to reduction in parasitism *ex vivo* as well as *in vivo*, and suppression of either compromises the macrophage-leishmanicidal activity [61–62]. IFN-γ is known to stimulate iNOS2 expression with significant NO secretion, that may have provided impressive levels of protection. In our studies, the antagonistic effect of IFN-γ in reducing Th2-associated cytokines, IL-10 and IL-4 was also found in treated mice. Detailed immunological analysis of infected mice depicted persistent IgG1 levels, probably maintained by IL-4 secretion [63]. Treatment with EE was associated with increased IgG2a, possibly due to up-regulated serum levels of IFN-γ. Plant-derived natural products have been found to exert leishmanicidal activity via modulation of the Th1/Th2 responses. Quassin inhibited *L*. *donovani* growth by switching from Th2 (IL-4 and IL-10) to Th1 (IFN-γ and IL-2) cytokine [21]. A similar effect occurred with EE, which enhanced IFN-γ and NO production, coupled with marked reduction in the *L*. *donovani* load in infected macrophages.

EE induced a marked switch in *L*. *donovani*-infected BALB/c mice from disease-promoting to *Leishmania*-specific disease-resolving humoral as well as cell-mediated immune responses. EE at 75mg/kg b.w. rescued T-cell-anergic conditions, inducing elevated levels of DTH, lymphoproliferation, IL-2, IFN-γ and NO, and maximally reduced the Th1 suppressive cytokines (IL-4 and IL-10), in a dose-dependent manner, whereas the immune response was restored following treatment with AMB (5mg/kg/b.w).

VL is associated with impaired immunological responses. Effectual antileishmanial therapy entails robust cellular immune responses in addition to antibodies. Experimental models of VL have shown that CD8^+^ T cells are instrumental in controlling *L*. *donovani*/*L*. *infantum* infection, through their ability to secrete IFN-γ and/or their cytolytic activity [64]. Furthermore, CD8^+^ coupled with CD4^+^ T cells, are essential to thwart reactivation of murine VL, the present investigation showed that the splenic CD4^+^ and CD8^+^ T lymphocyte counts were significantly boosted by administration of EE (75mg/kg b.w.) to *L*. *donovani* infected BALB/c mice as compared with untreated infected controls. The therapeutic significance of EE in VL was strengthened by the development of central memory (CD62L^high^ CD44^high^) CD8^+^ T lymphocytes.

T cells are known to interact with APCs and play vital roles in eliminating pathogens resident within macrophages. For optimal activation of naive T cells, the T-cell receptor (TCR) interacts with the peptide-MHC complex presented by professional APCs, while the second signal is delivered by co-stimulatory molecules, and the secretion of pro-inflammatory cytokines [65]. The ligation of CD80 and/or CD86 on APCs with CD28 on T cells induces the DCs to secrete IL-6 and IFN-γ for optimal T and B cell activation, proliferation, and differentiation [66]. There are reports indicating down modulation of CD80 and 86 expression in certain diseases [67–68], including leishmaniasis. In our studies, the expression of both CD80 and CD86 on peritoneal macrophages of *L*. *donovani* infected mice were found to be significantly enhanced upon treatment with EE, substantiating their immunomodulatory potency. Our results indicate the potentiality of EE in activating the T cells through upregulation of co-stimulatory signals that help to mount an effective immune response by secreting Th1 cytokines such as IFN-γ and IL-2.

The deterioration in renal and hepatic functions is the major dose-limiting side effect of currently available chemotherapeutic drugs. In the present study, upon administration of EE (25, 50 and 75mg/kg b.w.) to naïve and *L*. *donovani* infected BALB/c mice, the serum levels of SGOT, SGPT, ALP, urea and creatinine were found to be in the normal range, proving absence of hepato- and nephro-toxicity.

**Fig 10:**
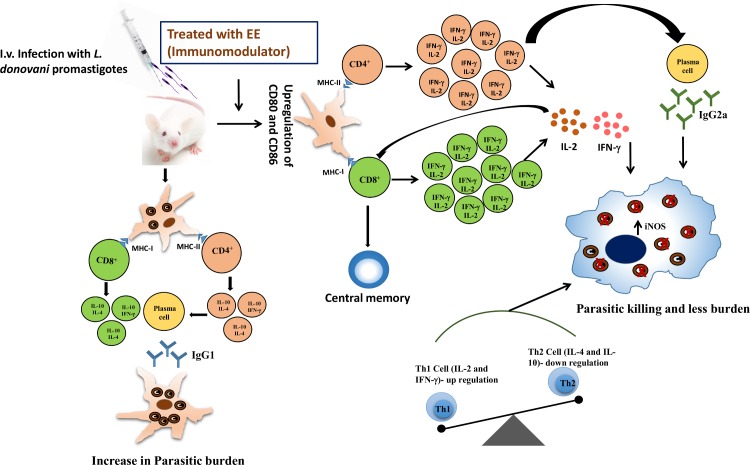
Proposed mechanism for immunotherapeutic potential of eugenol emulsion (EE).

In conclusion, our findings highlight the *in vitro* and *ex vivo* leishmanicidal effect of EE that occurred via an increase in production of NO without adverse effects on mammalian macrophages. On the other hand, treatment of infected mice with EE significantly waned the hepatic and splenic parasite loads with diminution in spleen and liver weights. EE induced enhanced lymphoproliferation, up-regulated co-stimulatory molecules (CD80/CD86) expression on APCs, and resulted in expansion of CD4^+^ and CD8^+^ T cell numbers and generation of central memory cells. EE further suppressed Th2 cytokines (IL-4 and IL-10) and stimulated the production of Th1 cytokines (IFN-γ and IL-2) with release of NO from the peritoneal macrophages. Th1-driven immune polarization was also reflected from high IgG2a/low IgG1 ratio and significant elicitation of DTH response. Thus, protection against *L*. *donovani* infection in the EE treated animals was due to direct parasite killing as well as the induction of cellular immunity via immunopotentiation as depicted in **Fig 10**.
